# Capping four years of growth of Molecular Autism: impact factor coming in 2014

**DOI:** 10.1186/2040-2392-4-50

**Published:** 2013-12-16

**Authors:** Joseph D Buxbaum, Simon Baron-Cohen

**Affiliations:** 1 Seaver Autism Center for Research and Treatment, Departments of Psychiatry, Neuroscience, and Genetics and Genomic Sciences, Friedman Brain Institute and Mindich Child Health and Development Institute, Icahn School of Medicine at Mount Sinai, New York, NY 10029, USA; 2 Autism Research Centre, Department of Psychiatry, University of Cambridge, Cambridge CB2 8AH, UK

## Editorial

We are pleased to announce that *Molecular Autism* has been accepted by Thomson Reuters for tracking and is due to receive its first official Impact Factor in June 2014. Since its inception in 2010, *Molecular Autism* has grown significantly, with the number of published articles increasing from 15 in 2010 to more than 45 in 2013. Since its launch, *Molecular Autism* has published over 95 articles. Many of our publications are highly accessed and highly cited, which has contributed to a very strong interest in the journal and submissions are steadily increasing. We have published excellent research across all aspects of autism, in keeping with our broad focus on basic, translational, and clinical research relevant to the etiology, pathobiology, and/or treatment of autism and related neurodevelopmental conditions.

We have attempted to ensure a rapid peer-review process such that articles are published in a timely manner; even with our stringent review process, the average time from submission to the first decision is less than 6 weeks. As an open access journal, all articles are immediately and freely available, contributing to the increase in access and readership; before the end of 2013 we had an average of 20,000 accesses per month, and November 2013 saw a record of 33,000 accesses. We are especially pleased that families who have a relative with autism or a related neurodevelopmental condition can access all articles in *Molecular Autism* free of charge. We are also pleased that clinicians and researchers worldwide, who may not have the resources afforded by major universities, can access all articles at no cost.

Links to publications are now listed on the *Molecular Autism* Twitter account and authors can obtain statistics about their articles, such as how many times these have been accessed and discussed on social media websites. These statistics can be retrieved by selecting ‘Article metrics’ in the right-hand column of each article.

We would like to thank our distinguished international Editorial Board for their efforts on behalf of the journal, and our publisher, BioMed Central, for their in-house contribution to the speed and efficiency with which manuscripts are processed. Most importantly, we thank our authors and our hard-pressed reviewers.

We invite scientists to continue to submit empirical and review articles of the highest standard, either reporting molecular data related to autism, or at other neural, cognitive or clinical levels, but where there may be some relevance for the overarching goal, which is to better understand the causes of autism and how best to support individuals with this diagnosis.

